# An Improved Genetically Encoded Fluorescent cAMP Indicator for Sensitive cAMP Imaging and Fast Drug Screening

**DOI:** 10.3389/fphar.2022.902290

**Published:** 2022-05-12

**Authors:** Wenfeng Liu, Chang Liu, Pei-Gen Ren, Jun Chu, Liang Wang

**Affiliations:** ^1^ Guangdong Provincial Key Laboratory of Biomedical Optical Imaging Technology & Center for Biomedical Optics and Molecular Imaging & CAS Key Laboratory of Health Informatics, Shenzhen Institute of Advanced Technology, Chinese Academy of Sciences, Shenzhen, China; ^2^ Center for Energy Metabolism and Reproduction, Shenzhen Institute of Advanced Technology, Chinese Academy of Sciences, Shenzhen, China; ^3^ Shenzhen College of Advanced Technology, University of Chinese Academy of Sciences, Shenzhen, China; ^4^ Shenzhen Institute of Synthetic Biology, Shenzhen Institute of Advanced Technology, Chinese Academy of Sciences, Shenzhen-Hong Kong Institute of Brain Science, Shenzhen, China

**Keywords:** single-FP indicator, fluorescence imaging, circular permutation, cAMP, GPCR, drug discovery, high-content screening

## Abstract

Cyclic adenosine 3′,5′-monophosphate (cAMP) is an important intracellular second messenger molecule downstream of many G protein-coupled receptors (GPCRs). Fluorescence imaging with bright and sensitive cAMP indicators allows not only dissecting the spatiotemporal dynamics of intracellular cAMP, but also high-content screening of compounds against GPCRs. We previously reported the high-performance circularly permuted GFP (cpGFP)-based cAMP indicator G-Flamp1. Here, we developed improved G-Flamp1 variants G-Flamp2 and G-Flamp2b. Compared to G-Flamp1, G-Flamp2 exhibited increased baseline fluorescence (1.6-fold) and larger fluorescence change (ΔF/F_0_) (1,300% vs. 1,100%) in HEK293T cells, while G-Flamp2b showed increased baseline fluorescence (3.1-fold) and smaller ΔF/F_0_ (400% vs. 1,100%). Furthermore, live cell imaging of mitochondrial matrix–targeted G-Flamp2 confirmed cytosolic cAMP was able to enter the mitochondrial matrix. G-Flamp2 imaging also showed that adipose tissue extract activated the Gi protein-coupled orphan GPCR GPR50 in HEK293T cells. Taken together, our results showed that the high-performance of G-Flamp2 would facilitate sensitive intracellular cAMP imaging and activity measurement of compounds targeting GPCR-cAMP signaling pathway during early drug development.

## Introduction

Cyclic adenosine 3′,5′-monophosphate (cAMP) is an important second messenger downstream of G protein-coupled receptors (GPCRs) that are coupled to Gs, Gi and Gq proteins (Massengill et al., 2021). It is produced from adenosine triphosphate (ATP) by adenylyl cyclases (ACs) and degraded to adenosine monophosphate (AMP) by phosphodiesterases (PDEs) (Cooper, 2003). cAMP signaling regulates a variety of cellular functions including cell migration, mitochondrial homeostasis, cell proliferation and cell death (Lefkimmiatis and Zaccolo, 2014). Given that cAMP can integrate signals from a diversity of GPCRs and its abnormal dynamics are associated with many diseases including cancer (Gold et al., 2013), the GPCR-cAMP signaling pathway is an attractive pharmaceutical target (Zaccolo, 2021).

The cAMP concentration is precisely controlled in space and time in living cells. cAMP and cAMP signaling could be constrained on or within subcellular structures (Zaccolo et al., 2021). Recently, cAMP and cAMP signaling in the mitochondrial matrix has gained much attention due to its key roles in regulating oxidative phosphorylation (OXPHOS) and mitochondrial biogenesis (Zhang et al., 2016). To measure the cAMP dynamics with high spatiotemporal resolution in mitochondrial matrix, optical imaging with genetically encoded fluorescent indicators (GEFIs) (Mattiazzi Usaj et al., 2016) is highly desirable.

During the past 20 years, more than 50 fluorescent protein (FP)-based indicators have been developed (Massengill et al., 2021). The majority of them are fluorescence resonance energy transfer (FRET) indicators and some are single-FP indicators. The latter usually gives much larger fluorescence change than the former (Massengill et al., 2021). However, current cAMP single-FP indicators (Flamindo2, cAMPr, Pink Flamindo and R-FlincA) exhibit small fluorescence change (|ΔF/F_0_| < 150%) and most are dim in mammalian cells at 37°C (Odaka et al., 2014; Harada et al., 2017; Hackley et al., 2018; Ohta et al., 2018). To address these problems, we recently developed a high-performance single-FP cAMP indicator named G-Flamp1 (Wang et al., 2022), which is created by inserting a circularly permuted GFP (cpGFP) into a bacterial cAMP-binding domain of the bacterial *Mloti*K1 channel (mlCNBD). G-Flamp1 exhibits a large fluorescence increase (a maximum ΔF/F_0_ of 1,100% in HEK293T cells), appropriate affinity (a *K*_*d*_ of 2.17 μM) and sub-second response kinetics. However, compared to the well-known single-FP calcium indicator GCaMP6s, the brightness of G-Flamp1 is still relatively low, which could hinder the cAMP imaging of subcellular structures such as mitochondria (Wang et al., 2022).

To further increase the signal-to-background ratio and sensitivity for cAMP imaging here we aimed to increase the brightness and dynamic range of the G-Flamp1 indicator. Using a combination of crystal structure-guided mutagenesis and library screening, we developed two variants named G-Flamp2 and G-Flamp2b with improved properties. Specifically, compared to G-Flamp1, G-Flamp2 exhibited an increased brightness (1.6-fold) and larger maximum ΔF/F_0_ (1300% vs. 1100% of G-Flamp1), while G-Flamp2b exhibited higher brightness (3.1-fold) but smaller ΔF/F_0_ (400% vs. 1100% of G-Flamp1) in HEK293T cells at 37°C. Then we demonstrated that G-Flamp2 enables sensitive monitoring of cAMP changes in mitochondrial matrix. Finally, based on G-Flamp2 imaging, we found that adipose tissue extracts can activate the Gi-coupled orphan GPCR GPR50. Taken together, these results suggest that G-Flamp2 is a powerful tool for sensitive cAMP imaging in subcellular compartments and image-based high-content screening (HCS).

## Results

### Development of G-Flamp2 and G-Flamp2b

**FIGURE 1:**
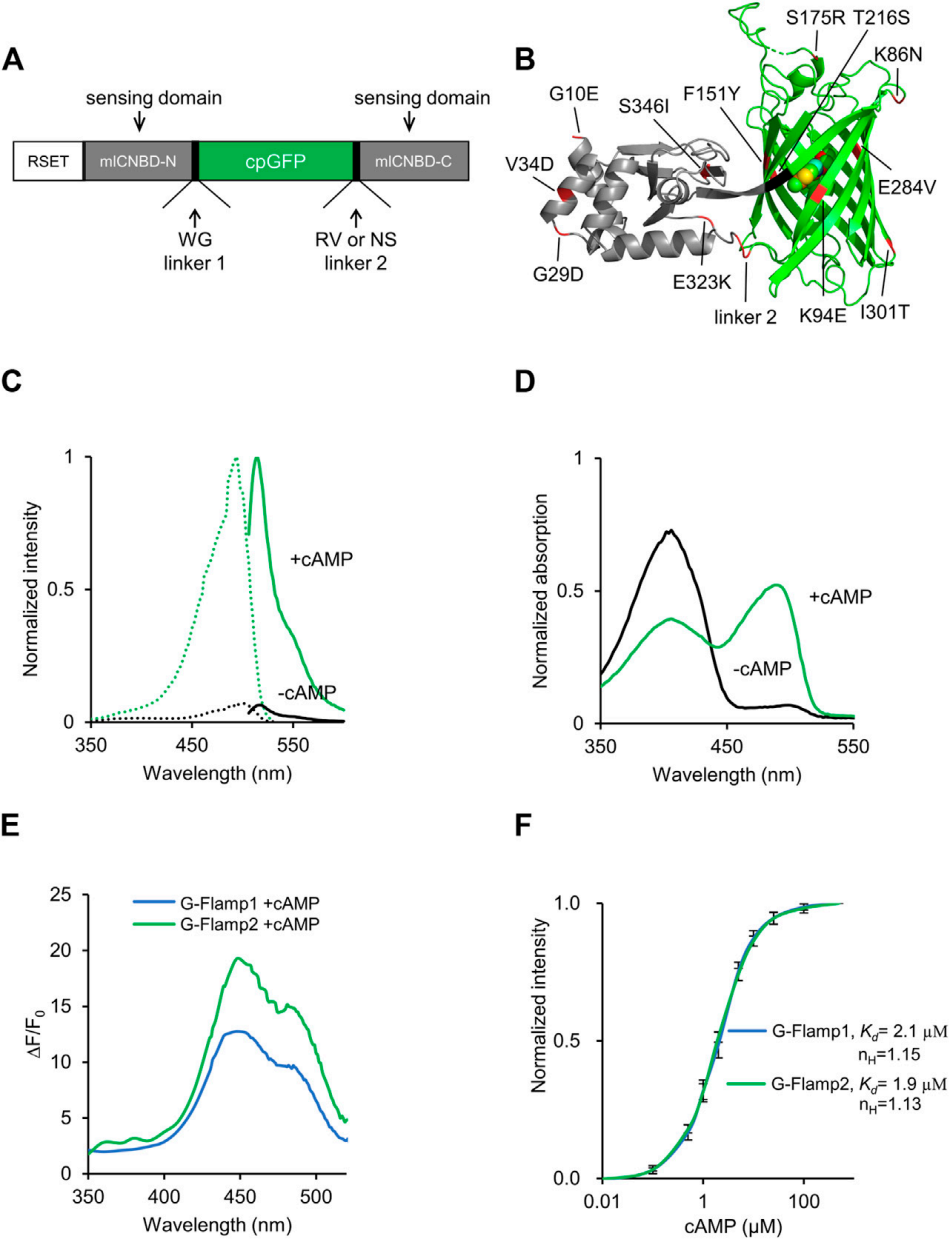
Development and *in vitro* characterization of G-Flamp2. **(A)** Schematic of G-Flamp biosensor. cpGFP was inserted into mlCNBD with linker1 and linker 2 (both have two amino acids). RSET: the N-terminal fusion peptide including a 6× His tag from the pNCS bacterial expression vector. **(B)** Crystal structure of G-Flamp1 (PDB 6M63). Sites mutated in G-Flamp2 and G-Flamp2b are marked in red. Linker is marked in black. **(C)** Excitation (dotted lines) and emission (solid lines) spectra of purified G-Flamp2 sensor in HEPES buffer (pH 7.15) with (green line, +cAMP) or without (black line, -cAMP) 500 μM cAMP. **(D)** Absorbance spectra of 50 μM G-Flamp2 in HEPES buffer (pH 7.15) in the presence (green line, +cAMP) or absence (black line, -cAMP) of 500 μM cAMP. **(E)** Excitation wavelength-dependent ΔF/F_0_ of G-Flamp1 and G-Flamp2 in HEPES buffer (pH 7.15) in the presence of 500 μM cAMP. **(F)** Binding titration curves of G-Flamp1 and G-Flamp2 biosensors to cAMP (500 μM) in HEPES buffer (pH 7.15). The data were fitted by a sigmoidal binding function to extract the dissociation constant *K*_*d*_. Data are presented as mean ± standard deviation (SD) from three independent experiments.

The cpFP cAMP indicator G-Flamp1 was originally constructed by inserting cpGFP from GCaMP6f into mICNBD (Nimigean et al., 2004; Mukherjee et al., 2016) at the mouth of the cAMP-binding pocket (Figure 1A). To develop improved G-Flamp1 variants, we focused on both the cpGFP and the interface between cpGFP and mlCNBD. First, we incorporated beneficial mutations from the OPT (Optimized) variant of circularly permuted super folder GFP (K86N/S175R/T216S/E284V/I301T, numbering according to PDB 6M63) into G-Flamp1 and generated G-Flamp1.5 variant with brightness increased by 17% (Figure 1B, Supplementary Figures S3A, S6). Second, we performed random mutagenesis on cpGFP part of G-Flamp1.5 using error-prone PCR and were able to identify a brighter (increased by 49%) (Supplementary Figure S3B) and highly responsive (ΔF/F_0_ of 1810%) variant G-Flamp2 (Figure 1B, Supplementary Figures S1A, S6), which harbors K94E mutation in the cpGFP. Inspection of crystal structure of G-Flamp1 revealed that the positive residue of Arg96 with the negative residue of Glu94 formed electrostatic interaction, and thus stabilized the protein structure (Supplementary Figure S4A). Third, to further increase the brightness of G-Flamp2, crystal structure–guided mutagenesis for stabilization of the interface between cpGFP and mlCNBD and random mutagenesis of mlCNBD were employed. The improved variant G-Flamp2b with brightness increased by 45% (Supplementary Figure S3C) was finally identified and has the following mutations: F151Y/E323K/S346I in the interface (Supplementary Figure S4B), G10E/G29D/V34D in mlCNBD and R318N/V319S in linker 2 (Figure 1B, Supplementary Figure S6). G-Flamp2b had a relatively small ΔF/F_0_ of 440% (Supplementary Figure S1B).

### Characterization of G-Flamp2 and G-Flamp2b *In Vitro*

We first investigated the fluorescence and absorption properties of purified G-Flamp2. The fluorescence excitation and emission spectra of cAMP-bound G-Flamp2 were similar to those of G-Flamp1 and peaked at 488 and 516 nm, respectively (Figure 1C). The excitation and emission peaks of cAMP-free G-Flamp2 were redder than those of cAMP-bound G-Flamp2 by 8 and 3 nm, respectively (Supplementary Table S1). G-Flamp2b had similar fluorescence spectra to those of G-Flamp2 (Supplementary Figure S2A). According to these fluorescence spectra, the calculated fluorescence change peaked at 450 nm with a maximum ΔF/F_0_ of 2000 and 430% for G-Flamp2 and G-Flamp2b, respectively (Figure 1E, Supplementary Figure S2B). Absorbance spectra of G-Flamp2 revealed that both cAMP-bound and cAMP-free indicators displayed two peaks with maxima at 400 and 490 nm. Moreover, the deprotonated form of cAMP-bound G-Flamp2 significantly increased, making it much brighter than deprotonated cAMP-free G-Flamp2 (Figure 1D).

The extinction coefficient (EC) of G-Flamp2 increased by 8.8-fold (5,189 mM^−1^cm^−1^ vs. 45,459 mM^−1^cm^−1^) after cAMP binding, while G-Flamp2b increased by 2.7-fold (12,528 mM^−1^cm^−1^ vs. 33,760 mM^−1^cm^−1^) (Supplementary Table S1). The quantum yield (QY) of G-Flamp2 and G-Flamp2b slightly decreased (0.38 vs. 0.33 for G-Flamp2 and 0.39 vs. 0.37 for G-Flmap2b) after cAMP binding (Supplementary Table S1). Like other single-FP probes, the fluorescence intensity of G-Flamp2 was sensitive to pH with pKa values of 8.50 and 6.97 for cAMP-free and cAMP-bound G-Flamp2, respectively (Supplementary Figure S7A, Supplementary Table S1). Moreover, the calculated ΔF/F_0_ peaked at pH 6.5 with a value of 1940% and remained high at pH 7.0 with a value of 1,590% (Supplementary Figure S7B), indicating that G-Flamp2 would be highly responsive in the pH environment of mammalian cells. Besides, the G-Flamp2b calculated ΔF/F_0_ peaked at pH 6.7 with a value of 370% and was still high at pH 7.0 with a value of 347% (Supplementary Figure S7C).

Next we examined the concentration-response relationship of G-Flamp2 and G-Flamp2b with cAMP or cGMP. The apparent dissociation constant (*K*_*d*_) values of G-Flamp2 and G-Flamp2b for cAMP were 1.9 and 3.2 μM, respectively (Figure 1F, Supplementary Table S1). The apparent *K*_*d*_ values of G-Flamp2 and G-Flamp2b for cGMP were 43 and 32 μM, respectively (Supplementary Figure S5). Based on the *K*_*d*_ values for cAMP and cGMP, G-Flamp2 and G-Flamp2b have ∼23 and 10-fold higher selectivity for cAMP over cGMP (defined as *K*_*d*_ ratio of cGMP/cAMP), while G-Flamp1 has a ∼13-fold selectivity towards cAMP (Ohta et al., 2018). This specificity is similar or better than most reported cAMP indicators (Supplementary Table S2). Since previous studies reported that cAMP indicators with *K*_*d*_ between 30 nM and 10 μM successfully monitored physiological cAMP changes (Börner et al., 2011; Kitaguchi et al., 2013; Odaka et al., 2014; Mukherjee et al., 2016; Ohta et al., 2018), G-Flamp2 and G-Flamp2b would be well suitable for most applications.

### Performance of G-Flamp2 and G-Flamp2b in HEK293T Cells

**FIGURE 2:**
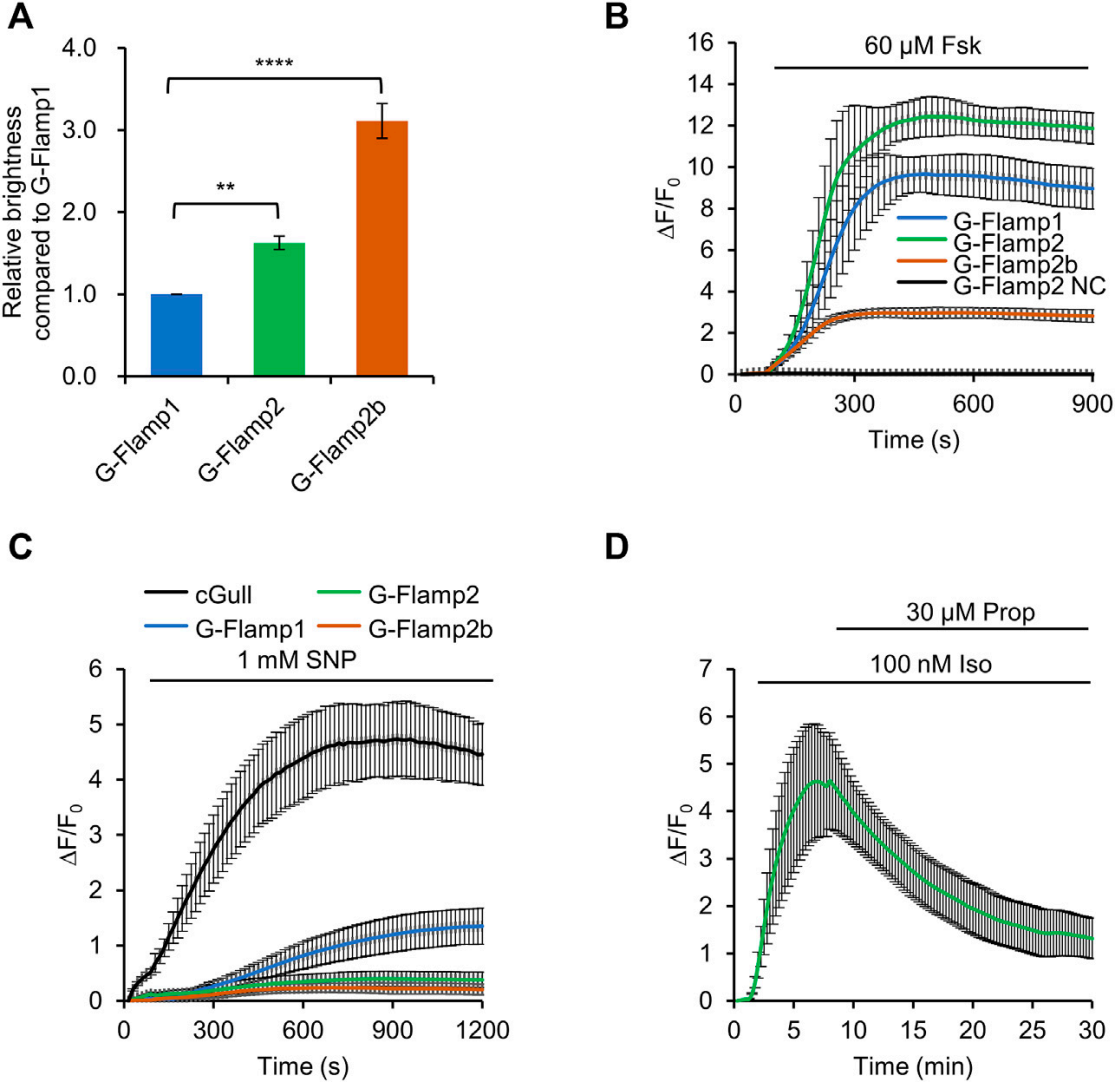
Characterization of G-Flamp2 and G-Flamp2b in HEK293T cells. **(A)** Relative brightness of G-Flamp1, G-Flamp2 and G-Flamp2b in resting HEK293T cells measured using a plate reader (excited at 450 nm). The IRES-mCherry-CAAX gene was attached downstream of the green sensors and the red fluorescence intensity was used to calibrate the sensor’s brightness. **(B)** Traces of ΔF/F_0_ in responses to 60 µM Fsk of G-Flamp1, G-Flamp2, G-Flamp2b and G-Flamp2 NC (G-Flamp2 R341E, cAMP-insensitive negative control) in HEK293T cells. Data are presented as mean ± standard deviation (SD) from three independent experiments. *n* = 7, 13, 12, 9 cells for G-Flamp1, G-Flamp2, G-Flamp2b and G-Flamp2 NC, respectively. **(C)** Similar to **(B)** except that 1 mM SNP was used. *n* = 15, 12, 10 cells for G-Flamp1, G-Flamp2 and G-Flamp2b, respectively. *n* = 5 cells for cGull. **(D)** Traces of ΔF/F_0_ in response to 100 nM Iso followed by 30 μM propranolol in HEK293T cells expressing G-Flamp2. *n* = 16 cells from 3 cultures. Data are presented as the mean ± standard deviation. *Significantly different from G-Flamp1, **p < 0.01, ****p < 0.0001 as measured by One-way analysis of variance (ANOVA) with post hoc Tukey’s test.

We first examine the fluorescence brightness of G-Flamp1/2/2b indicators in HEK293T cells. Under 450 nm or 480 nm excitation, G-Flamp2 and G-Flamp2b showed a 1.6-fold and 3.1-fold higher baseline fluorescence than G-Flamp1, respectively (Figure 2A, Supplementary Figure S8).

Then we investigated the fluorescence change of G-Flamp1/2/2b sensors. To this end, 60 μM Forskolin (Fsk), a potent pharmacological activator of transmembrane adenylyl cyclase (tmAC), was used to induce a large amount of cAMP in HEK293T cells. Under 450 nm excitation, G-Flamp1, G-Flamp2 and G-Flamp2b exhibited a maximum ΔF/F_0_ of 1,000, 1200 and 300%, respectively (Figure 2B). As a negative control, the cAMP-insensitive G-Flamp2 NC (G-Flamp2 Negative Control) sensor with the R341E mutation showed no detectable fluorescence change (Figure 2B).

Next we characterized the specificity and reversibility of G-Flamp1/2/2b indicators. We first tested the responses of indicators to cGMP, which was reported to bind the mlCNBD with a weaker affinity than cAMP. 1 mM sodium nitroprusside (SNP, a NO donor that activates soluble guanylyl cyclase) was exploited to induce a large amount of cGMP in HEK293T cells. Under 480 nm excitation, Green cGull, a single fluorescent protein (FP)-based cGMP indicator, exhibited a large fluorescence change with a peak ΔF/F_0_ of 500% (Figure 2C) (Ponsioen et al., 2004; Matsuda et al., 2017). Under 450 nm excitation, G-Flamp1 exhibited a significant fluorescence increase with a peak ΔF/F_0_ of 134% (Figure 2C), while G-Flamp2 and G-Flamp2b showed a relatively smaller peak ΔF/F_0_ (40 and 20%, respectively) (Figure 2C). These results indicated that G-Flamp2/2b had a high specificity toward cAMP. Regarding reversibility, HEK293T cells expressing G-Flamp2 exhibited increased fluorescence upon 100 nM Iso treatment and then returned to basal level after addition of 30 μM β-AR anti-agonist propranolol (Prop) (Figure 2D).

Taken together, the above results demonstrated that both G-Flamp2 and G-Flamp2b had higher brightness and better specificity than G-Flamp1, and G-Flamp2 also had a larger fluorescence change than G-Flamp1 in living mammalian cells. Therefore, G-Flamp2 is more suitable to monitor intracellular cAMP dynamics.

### Cytosolic cAMP Enters Into Mitochondrial Matrix

The origin of cAMP in the mitochondrial matrix is still under debate (Valsecchi et al., 2013). Since cAMP can not freely diffuse across the inner mitochondrial membrane (Acin-Perez et al., 2009), one model suggested that cAMP inside mitochondria is actively transported from cytoplasmic pool (DiPilato et al., 2004) and another one suggested that the intramitochondrial cAMP is produced by mitochondrially localized soluble AC (sAC) (Zhang et al., 2016).

**FIGURE 3:**
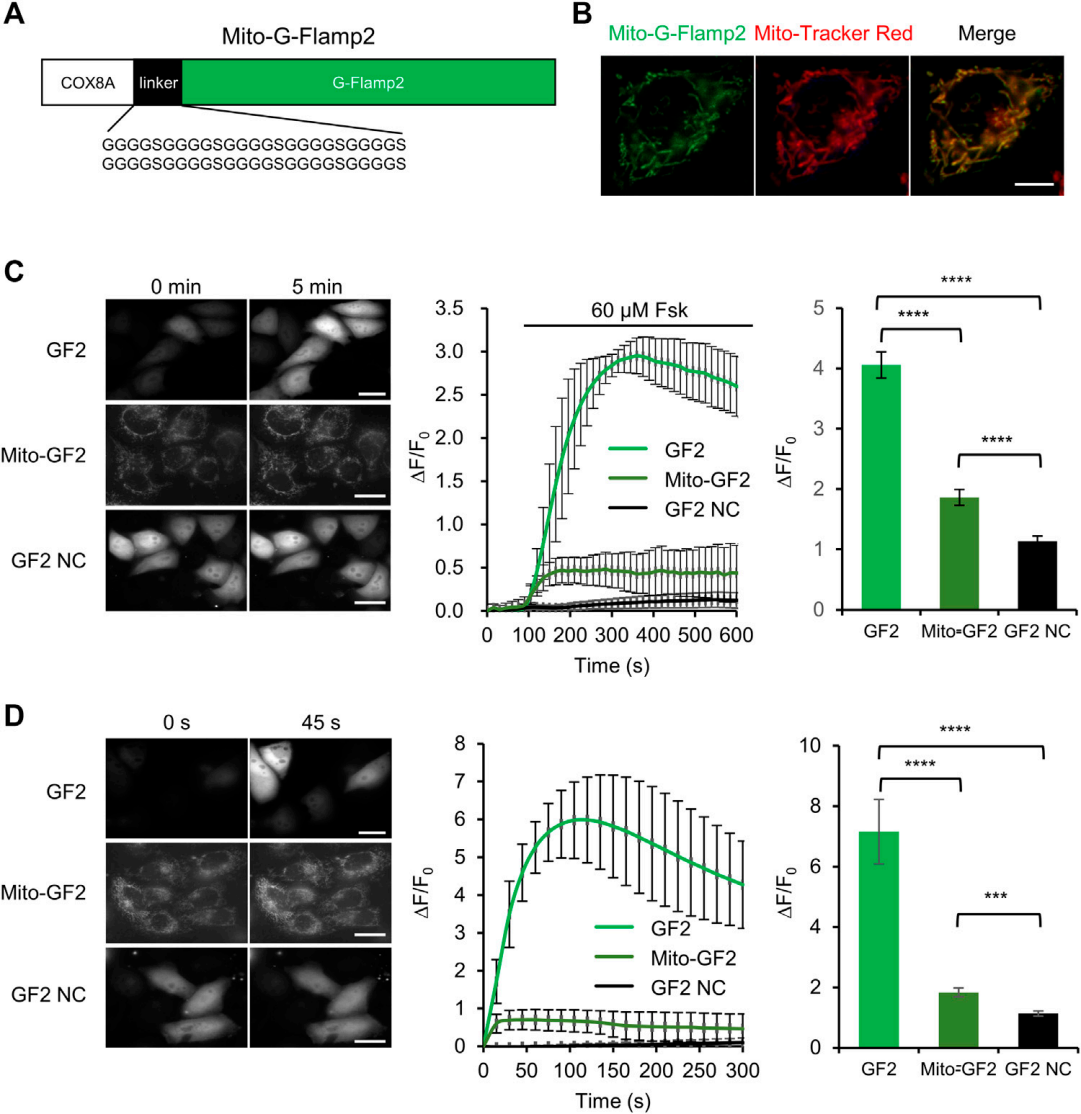
cAMP diffusion from cytosol to mitochondrial matrix in HeLa cells. **(A)** Schematic of mitochondrial matrix-localized G-Flamp2 (Mito-G-Flamp2) biosensor. The mitochondrial-targeting sequence from subunit VIII of the human cytochrome oxidase (COX) was fused to the N-terminus of G-Flamp2 with a flexible (G4S)_10_ linker. **(B)** Epifluorescence images of Mito-G-Flamp2 (Green) and Mito-Tracker (Red) in HeLa cells. (Scale bars, 5 μm). **(C)** Epifluorescence images of G-Flamp2 (GF2), Mito-G-Flamp2 (Mito-GF2) and G-Flamp2 NC (GF2 NC) to 60 µM Fsk stimulation in HeLa cells. Response of GF2, Mito-GF2 and GF2 NC to 60 µM Fsk stimulation. Quantification of peak ∆F/F_0_ are on the right. *n* = 11, 12 and 11 cells for GF2, Mito-GF2 and GF2 NC from 3 cultures for each sensor. (Scale bars, 10 μm). **(D)** Epifluorescence images of GF2, Mito-GF2 and GF2 NC to cAMP that is generated by bPACm in HeLa cells. The absorption peak of purified bPAC with and without light is at 453 and 441 nm, respectively, which is coincidentally consistent with the spectral-dependent G-Flamp sensors that the excitation peak at ∼ 450 nm. Response of GF2, Mito-GF2 and GF2 NC to cAMP that is generated by bPAC mutation. Quantification of peak ∆F/F_0_ are on the right. *n* = 12, 14 and 12 cells for GF2, Mito-GF2 and GF2 NC from 3 cultures for each sensor. (Scale bars, 10 μm). Data are presented as the mean ± standard deviation. *Significantly different from GF2 NC, ***p < 0.001, ****p < 0.0001 as measured by one-way analysis of variance (ANOVA) with post hoc Tukey’s test. See Supplementary Figure S9.

To test whether cytosolic cAMP can enter mitochondria, we monitor cAMP dynamics inside the mitochondrial matrix while stimulating cAMP production in cytosol. We fused mitochondrial matrix-targeting sequence from subunit VIII of the human cytochrome oxidase (COX) at the N terminus of G-Flamp2 to generate Mito-G-Flamp2 (Figure 3A). Mito-G-Flamp2 showed good colocalization with the mitochondria marker dye Mito-Tracker Red CMXRos (Figure 3B). HeLa cells expressing Mito-G-Flamp2, G-Flamp2 and G-Flamp2 NC showed a peak ΔF/F_0_ of 50, 260 and 10% after 60 μM Fsk treatment, respectively (Figure 3C, Supplementary Figure S9A–C). Since Fsk only activate tmAC but not sAC (Steegborn, 2014), these results suggests that cAMP generated in the cytosol can access the mitochondrial matrix and lead to the fluorescence increase of Mito-G-Flamp2.

A potent blue light-activatable optogenetic adenylyl cyclase bPACm (a bPAC variant with much lower dark activity) (Yang et al., 2021), was also used to specifically increase the cytosolic cAMP concentration. Mito-G-Flamp2, G-Flamp2 or G-Flamp2 NC were co-expressed with bPACm-mCherry in HeLa cells and the 450 nm light was exploited to activate bPACm and excite the indicators simultaneously. Mito-G-Flamp2, G-Flamp2 or G-Flamp2 NC exhibited a peak ΔF/F_0_ of 60, 590 and 20%, respectively (Figure 3D, Supplementary Figure S9D–F). These results confirmed that cytosolic cAMP can enter mitochondria.

### Adipose Tissue Extract Activates the Orphan GPCR GPR50 in HEK293T Cells

GPR50 is an orphan receptor of the GPCR family and its endogenous ligand has not been identified. It has been shown that GPR50 interacts with Gi and inhibits cAMP production in GPR50-overexpressing HEK293T cells (Levoye et al., 2006; Hand, 2012). Interestingly, GPR50 is down-regulated in breast, cervical, ovarian and lung cancers while up-regulated in liver cancer (Wojciech et al., 2018; Saha et al., 2020). Hence, GPR50-cAMP signaling pathway is a promising cancer drug target and screening ligands for GPR50 is of great importance.

**FIGURE 4:**
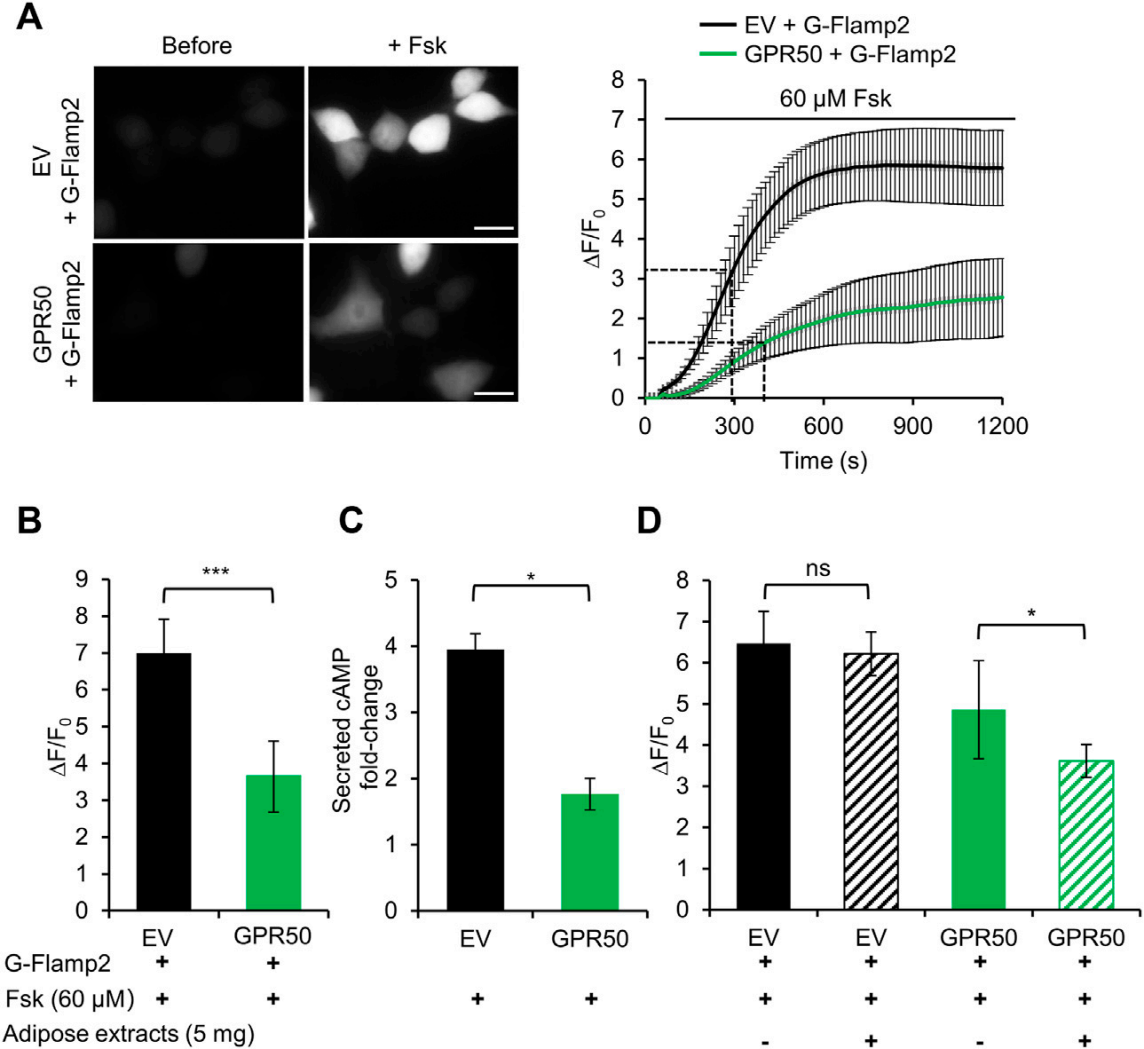
Adipose tissue extract activates the orphan GPCR GPR50 in HEK293T cells. **(A)** Representative fluorescence images (left) and traces of ΔF/F_0_ in response to 60 μM Fsk of G-Flamp2 in HEK239T cells stably expressing Empty Vector (EV) or GPR50 receptor (GPR50) (right). *n* = 14 and 10 cells for EV and GPR50, respectively. **(B)** Peak ΔF/F_0_ in response to 60 μM Fsk in HEK293T cells stably expressing EV or GPR50. **(C)** Quantification of intracellular cAMP levels in EV and GPR50 cells after 20 min in stimulation of 60 μM *n* = 3 cultures for both. **(D)** Peak ΔF/F_0_ in response to 5 mg/ml adipose extracts pretreatment for 5 min with 60 μM Fsk in HEK293T cells stably expressing EV or GPR50. Scale bars: 50 μm. Data are presented as the mean ± standard deviation (SD). *p < 0.05, ***p < 0.001 as measured by a Two-tailed Student’s *t*-test.

Here we take the advantage of high spatiotemporal resolution of G-Flamp2 imaging to explore the dynamics of cAMP production inhibition by overexpression of GPR50 and analyze the effect of adipose tissue extracts on GPR50 activation. We first established stable 293T cells overexpressing GPR50 using the lentivirus and confirmed the expression of GPR50 at the mRNA and protein levels (Supplementary Figure S10). Then we used G-Flamp2 sensor to detect the dynamics of cAMP in living HEK293T cells. Upon 60 μM Fsk stimulation, G-Flamp2 in GPR50-expressing HEK293T cells exhibited a smaller peak fluorescence increase (ΔF/F_0_ of 350% vs. 680%) and larger half-rise time (400 vs. 300 s) than in control cells (Figures 4A,B). Measurements of total cAMP in cell lysate using a commercial cAMP parameter assay kit confirmed that GPR50-expressing cells produced only half amount of cAMP in control cells after a 15 min treatment of 60 μM Fsk (Figure 4C). The above results using G-Flamp2 validate that GPR50 is Gi-coupled and provide the basis for subsequent screening of GPR50 ligands by G-Flamp2.

Next, we used this system to identify agonists or tissue fluid against GPR50. Our previous experiments demonstrated that GPR50 is highly expressed in adipose tissues (Yao et al., 2019) and adipose tissue extracts were tested here. G-Flamp2 in GPR50-expressing HEK293T cells with a 5 min pretreatment with adipose tissue extracts showed a smaller peak ΔF/F_0_ (360 vs. 485%) than in cells without pretreatment (Figure 4D), which suggested that GPR50 may be activated by certain compound(s) in the adipose tissue extract. To further identify the exact compound(s) activating GPR50, fractions separated by chromatography of adipose tissue extracts could be tested by G-Flamp2 imaging method. Taken together, the above results demonstrated that G-Flamp2 has high sensitivity in monitoring the inhibition effects of GPR50 on the total amount and rate of cAMP production and can be used to screen compounds against GPCR-cAMP signaling.

## Discussion

In this study, we engineered two G-Flamp1 variants G-Flamp2 and G-Flamp2b with improved brightness. G-Flamp2 also showed larger maximum ΔF/F_0_ than G-Flamp1 in HEK293T cells (ΔF/F_0_ ∼ 1,300% vs. 1,100%), which is also the largest among the cAMP fluorescent sensors reported. G-Flamp2/G-Flamp2b can be paired with spectrally distinct indicators (e.g. red calcium sensor) for multiplex imaging, since the single-FP indicator only occupy one fluorescence channel, which provides the possibility for simultaneous labeling and visualization of multiple molecule activities in living cells. Thus, G-Flamp2 and G-Flamp2b could be robust tools for basic cell biology research and early drug screening.

Using mitochondria-localized G-Flamp2, we observed increase of cAMP level in the mitochondrial matrix when cytosolic cAMP was increased by high concentration of Fsk or activated bPACm (a potent blue light-activatable AC) in HeLa cells, which is similar to the result in DiPilato et al. (DiPilato et al. 2004) and indicated that cytosolic cAMP can enter mitochondrial matrix. However, other studies such as Di Benedetto *et al* and Lefkimmiatis *et al* demonstrated that cAMP generated in the cytosol did not enter the mitochondrial matrix, except during mitochondrial permeability transition (Di Benedetto et al., 2013; Lefkimmiatis et al., 2013). These different observations might be due to different sensitivities of the indicators used (Wang et al., 2022). For those biological systems in which cytosolic cAMP can enter mitochondrial matrix, further studies are required to dissect the underlying mechanism.

Using the tumor-related Gi-coupled GPCR (GPR50) signaling pathway as a model, our G-Flamp2 living cell imaging experiments demonstrated that adipose tissue extract activated the orphan GPCR GPR50. These data indicated that G-Flamp2 could be a useful fluorescent probe for image-based high-content screening (HCS), which emerges as a powerful technique in both quantitative cell biology studies and drug discovery. Recently, the single-FP cAMP indicator cADDis was used to detect cAMP level changes upon the stimulation of the ligand of Gs or Gi protein-coupled GPCRs using automated microscope methods (Tewson et al., 2016; Clayton, 2018), suggesting its potential capabilities in HCS. Since G-Flamp2 has a higher affinity for cAMP (*K*_*d*_ of 1.9 μM vs. 10–100 μM) and larger fluorescence change (ΔF/F_0_ of 13 vs. −0.55) than cADDis (Klausen et al., 2019), and a relatively high brightness, G-Flamp2 would be more suitable for HCS to identify and evaluate the effect of candidate compounds targeting GPCR-cAMP signaling pathways in drug discovery.

## Methods

### Chemicals

cAMP-Na (Cat. No. A6885), cGMP-Na (Cat. No. G6129) and 3-isobutyl-1-methylxanthin (IBMX) (Cat. No. I5879) were purchased from Sigma-Aldrich (Darmstadt, Germany). cAMP (Cat. No. C107047), isoproterenol HCl (Cat. No. I129810) and propranolol (Cat. No. S133437) were purchased from Aladdin (Shanghai, China). Forskolin (Cat. No. S1612), puromycin (Cat. No. ST551), SNP (Cat. No. S0015), Mito-Tracker Red CMXRos (Cat. No. C1049B) and RIPA lysis buffer (Cat. No. P0013B) were purchased from Beyotime Biotechnology (Shanghai, China).

### Plasmid Constructions

bPAC mutation is a gift from Shiqiang Gao, who works at Department of Biology, Institute for Molecular Plant Physiology and Biophysics, Biocenter, ulius-Maximilians-University of Würzburg, Julius-von-Sachs-Platz 2, 97,082 Würzburg, Germany. Plasmids were made using the Infusion method (Takara Bio United States, Inc.). PCR fragments were amplified using PrimerStar (normal PCR or site-directed mutagenesis) or Taq (random mutagenesis) DNA polymerases. When needed, overlap PCR was exploited to generate the intact DNA fragment for Infusion. All PCR primers were purchased from Sangon Biotechnology Co., Ltd. (Shanghai, China). DNA sequence for Green cGull and mitochondrial targeting peptide was synthesized by Genscript. To express fluorescent proteins or sensors in bacterial or mammalian cells, cDNAs of FPs or sensors were subcloned into pNCS or pCAG vector (Chu et al., 2016). The pNCS and pCAG-mEGFP were kept in our lab. For mitochondrial localization of G-Flamp2, DNA coding sequences for mitochondrial targeting peptide of COX8A (G4S)10 and G-Flamp2 was overlapped and cloned into pCAG vector. All constructs were confirmed by DNA sequencing (Sangon Biotechnology Co., Ltd., Shanghai, China).

### Screening of cAMP Sensors Expressed in Bacteria

The DNA ligation products for site-directed or random mutagenesis were transformed into DH5α cells lacking adenylate cyclase gene CyaA(DH5α-ΔCyaA). After overnight growth at 34°C, the colonies with different fluorescence intensities on the LB agar plates were screened by eye in a BlueView Transilluminator (Vernier) with the 400–500 nm excitation light and a yellow acrylic long-pass filter, or by fluorescence imaging in a home-made imaging system with 480/20 nm excitation and 520/20 nm emission filters. To quantitatively compare the brightness of selected variants, bacterial patches on the agar plates cultured overnight at 34°C were imaged in the home-made system mentioned above and analyzed by ImageJ software (National Institutes of Health). The fluorescence change (ΔF/F_0_) of the cAMP sensor mutants were tested as previously described (Wang et al., 2022). Briefly, selected bacterial colonies were patched on LB agar plate and grew at 25°C for 3 days. Then the bacteria from each patch were collected, suspended in HEPES buffer (pH = 7.15) and lysed by sonication. The clear lysates were then used for measuring the ΔF/F_0_ in response to 500 μM cAMP with an Infinite M1000 PRO fluorometer (Tecan).

### Bacterial Protein Expression, Purification and Characterization

Expression, purification and characterization of G-Flamp biosensor with a N-terminal 6×His tag were performed as previously reported (Wang et al., 2022). Briefly, the sensors were transformed into DH5αΔCyaA cells and cultured on SOB agar plates for 3 days at room temperature. The bacterial cells were harvested and suspended in HEPES buffer and lysed by sonication. The proteins in the clear lysates were purified using HisPur Cobalt Resin (Pierce, Rockford, IL) and desalted with HEPES buffer using the gel filtration column (Bio-Rad).

The concentrations of purified sensors were measured by the BCA assay (Bio-Rad, Hercules, CA). Excitation and emission spectra were measured with an Infinite M1000 PRO fluorometer (Tecan). Extinction coefficients were calculated using the previously described ‘base denatured chromophore’ method (Chu et al., 2016). Quantum yields were determined using mEGFP as a standard (QY = 0.60). pH titrations were performed by mixing 10 μL concentrated protein solution with 110 μL buffers of different pH ranging from 2 to 11 and the pKa was determined as described previously (Wang et al., 2022). cAMP titrations were performed by mixing 1 μM of purified protein in HEPES buffer with varying concentrations of cAMP (0.001, 0.01, 0.1, 0.5, 1, 2, 5, 10, 25, 100 and 500 μM) or cGMP (0.01, 0.1, 0.5, 1, 2, 5, 10, 25, 100, 500, 1,000 and 2000 μM) and *K*_*d*_ and Hill coefficient were determined as described previously (Wang et al., 2022).

### Cell Culture and Transfection

HEK293T and HeLa cells were cultured in high glucose Dulbecco’s modified Eagle’s medium (Gibco, Grand Island, NY) supplemented with 10% (v/v) fetal bovine serum (Gibco, Grand Island, NY) and 100 units/mL penicillin/streptomycin (Gibco, Grand Island, NY) in a humidified incubator at 37°C with 5% CO2. Plasmid transfections of cultured cells on 6 or 12-well plates and glass-bottom coverslips were performed according to the manufacturer’s manuals of Lipofectamine 2000.

### Brightness Comparison of cAMP Indicators in HEK293T Cells

HEK293T cells grown in 12-well plates were transfected with pCAG-G-Flamp1-IRES-mCherry, pCAG-G-Flamp2-IRES-mCherry, pCAG-G-Flamp2b-IRES-mCherry construct separately using Lipofectamine 2000 48 h later, they were washed once with PBS, suspended in live cell imaging solution and transferred to a clear flat-bottom 96-well plate. The green and red fluorescence was recorded under 480 nm excitation and 560 nm excitation, respectively.

### Wide-Field Fluorescence Imaging of cAMP Indicators in Living Cells

Wide-filed fluorescence imaging was carried out on an Olympus IX83 microscope equipped with a 60 ×1.4 numerical aperture (NA) objective (for Figures 2, 3) or 40 ×1.25 NA objective (for Figure 4). Briefly, cells grown on glass-bottom dishes (Cat. No. #FD35-100, World Precision Instruments) were transfected with indicated plasmids and cultured for 24 h or 48 h. Then the cells were serum starved for 4 h and culture medium was replaced with live cell imaging solution (Cat. No. A14291DJ, Invitrogen) right before imaging. The excitation and emission filters used for different sensors or fluorescent proteins were as follows: ex 480/30 nm and em 530/30 nm for Green cGull, ex 568/20 nm and em 630/50 nm for mCherry, and ex 441/20 nm and em 530/30 nm for G-Flamp1, G-Flamp2 and G-Flamp2b. Images were acquired every 15 s for Forskolin stimulation (for Figures 2, 3C, 4) and 5 s for mitochondria-targeted indicators (for Figure 3D).

### Generation of HEK293T Cell Lines Stably Overexpressing GPR50

Lentiviruses were used for establishing of stable cell lines expressing GPR50. Briefly, HEK293T cells were seeded into 6-well plates at a density of 1 × 10^6^ cells/well and cultured for 24 h, infected with PLV7-GPR50 lentivirus and or PLV7 (control) lentivirus separately for 24 h and then replaced with normal medium. 72 h later, medium containing 2 μg/ml puromycin was used for stable cell line selection.

### Adipose Tissue Extract Preparation

0.5 g epididymal adipose tissue from 8-week old male mice was washed with cold PBS containing gentamicin (50 mg/L) three times. Then the tissue was cut into small pieces and homogenized to grind the tissue. Lipid components were extracted with Soxhlet extractor (Palmisciano Bedê et al., 2015) and dissolved in DMSO.

### Quantitative Real-Time Polymerase Chain Reaction

Total RNA from cultured cells was extracted with TRIzol Reagent (Invitrogen, Carlsbad, CA). The qPCR reactions were performed according to the SYBR Premix Ex Taq II (Takara, Japan) protocol. RNA levels were calculated using the ΔΔCT method, where CT is the cycle threshold. The amount of β-actin mRNA was used as a reference. Forward and reverse primers for GPR50 were 5′, GCT​CCG​AAA​TTC​TGG​CAA​CAT and 5′TCA​AAG​GGT​AGG​GGT​AGA​TGG, respectively. Forward and reverse primers for β-actin were: 5′ GTA​TCC​ATG​AAA​TAA​GTG​GTT​ACA​GG and 5′GCA​GTA​CAT​AAT​TTA​CAC​AGC​AAT, respectively.

### Immunofluorescence

GPR50-293T or Empty Vector-293T cells cultured on coverslips for 24 h were washed with PBS twice and fixed with 4% paraformaldehyde solution. Plasma membranes were permeabilized with 1% Triton X-100 solution for 5 min and 5% Bovine Serum Albumin (BSA, Sangon Biotech, China) was used for blocking for 30 min. Cells were then incubated with diluted primary antibody against GPR50 (#19762-AP, Proteintech, China) at 4°C overnight. Cells were then washed with PBS for three times. Then cells were incubated with Alexa-488-Donkey anti-rabbit (A21206, Invitrogen, CA) for 2 h and stained by DAPI staining solution (Beyotime, China) for 5 min in the dark at room temperature. The fluorescence images were acquired with a Olympus IX71 microscope equipped with 20 × 1.25 NA objective.

### cAMP ELISA Assay

GPR50-293T or Empty Vector-293T cells were seeded into 6-well plates at a density of 1 × 10^6^ cells/well and cultured for 24 h. Then the cells were pre-treated with 200 μM IBMX for 30 min and then stimulated with 60 μM forskolin for another 30 min to induce cAMP production. Next the cells were washed with cold PBS three times, and the amount of cAMP in cells were measured using the Parameter cAMP assay kit (R&D Systems, United Kingdom).

### Data Analysis and Statistics

Analysis of the fluorescent images were performed using ImageJ software (NIH software). Background-subtracted fluorescence was used to calculate fluorescence change ΔF/F_0_ that is defined as (F-F_0_)/F_0_, where F_0_ is the baseline signal before stimulation. The statistical significances between groups were determined using the two-tailed unpaired Student’s *t*-test (Figure 4) or one-way ANOVA (Figures 2, 3) with GraphPad Prism 7.0 software (La Jolla, CA). *p < 0.05, **p < 0.01, ***p < 0.001 and NS (not significant) for *p* > 0.05.

## Data Availability

The raw data supporting the conclusions of this article will be made available by the authors, without undue reservation.
