# Supplementary material for: An Improved Genetically Encoded Fluorescent cAMP Indicator for Sensitive cAMP Imaging and Fast Drug Screening
Source: Front Pharmacol. 2022 May 12;13:902290. doi: 10.3389/fphar.2022.902290 (PMC9175130; doi:10.3389/fphar.2022.902290)
Supplement: Supplementary file 1 [file DataSheet1.pdf]

Supplementary Material

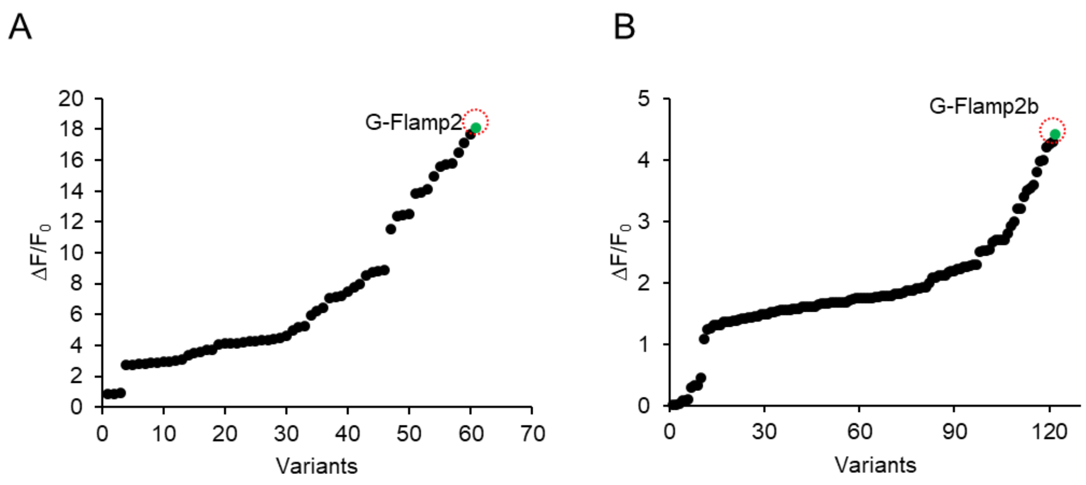

Supplementary Figure 1 Evolution of G-Flamp2 and G-Flamp2b.
(A) (B) $\Delta F/F_0$ of 61 and 122 variants of G-Flamp2 and G-Flamp2b with screening for linkers, sensing domains and cpGFP. The dynamic range ($\Delta F/F_0$) suggests the signal change of the supernatant of bacterial lysate in response to 500 μM cAMP. The variant with largest $\Delta F/F_0$ was named G-Flamp2 **(A)**, and the variant with medium $\Delta F/F_0$ but highest brightness was selected and called G-Flamp2b **(B)**.

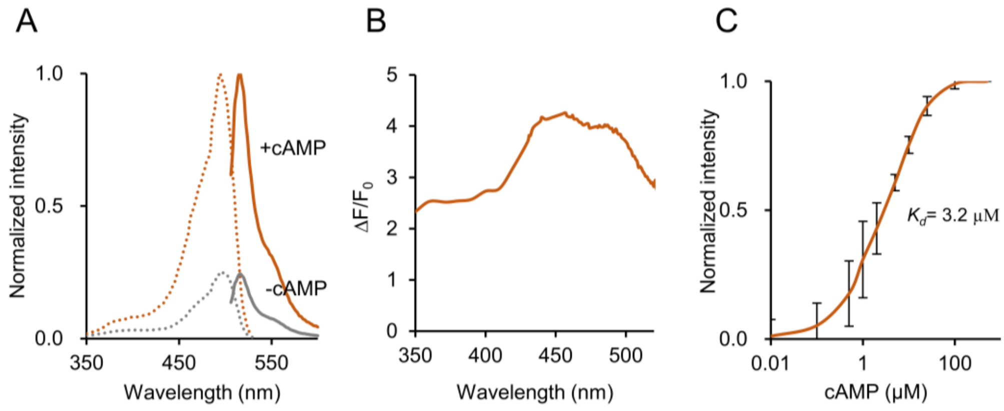

Supplementary Figure 2 Characterization of G-Flamp2b *in vitro*
(A) Excitation (dotted lines) and emission (solid lines) spectra of purified G-Flamp2b sensor in HEPES buffer (pH 7.15) with (orange line, +cAMP) or without (grey line, -cAMP) 500 μM cAMP.
(B) Excitation wavelength-dependent $\Delta F/F_0$ of G-Flamp2b in HEPES buffer (pH 7.15) in the presence of 500 μM cAMP.
(C) Binding titration curves of G-Flamp2b to cAMP (500 μM) in HEPES buffer (pH 7.15). The data were fitted by a sigmoidal binding function to extract the dissociation constant K_d. Data are presented as mean ± standard deviation (SD) from three

independent experiments.

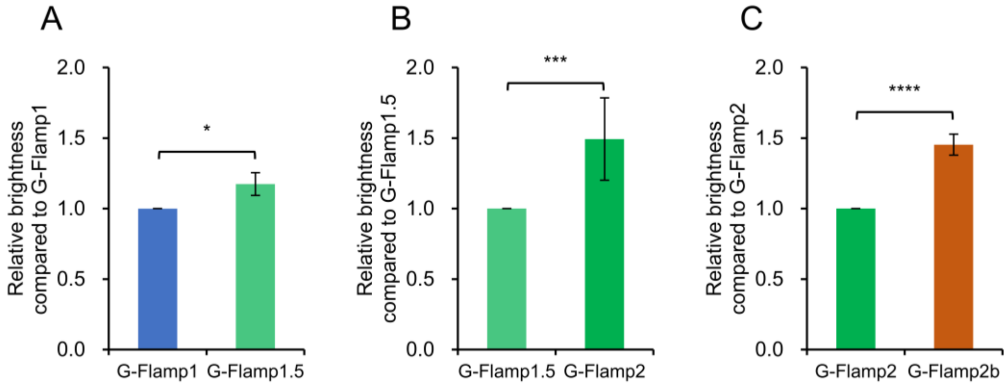

Supplementary Figure 3 The relative brightness of G-Flamp biosensors.

(A) The relative brightness of G-Flamp1 and G-Flamp1.5 in bacterial cells cultured overnight at 34°C.

(B) The relative brightness of G-Flamp1.5 and G-Flamp2 in bacterial cells cultured overnight at 34°C.

(C) The relative brightness of G-Flamp2 and G-Flamp2b in bacterial cells cultured overnight at 34°C.

Data are presented as the mean ± standard deviation. *$p < 0.05$, ***$p < 0.001$, ****$p < 0.0001$ as measured by Two-tailed Student's *t*-test.

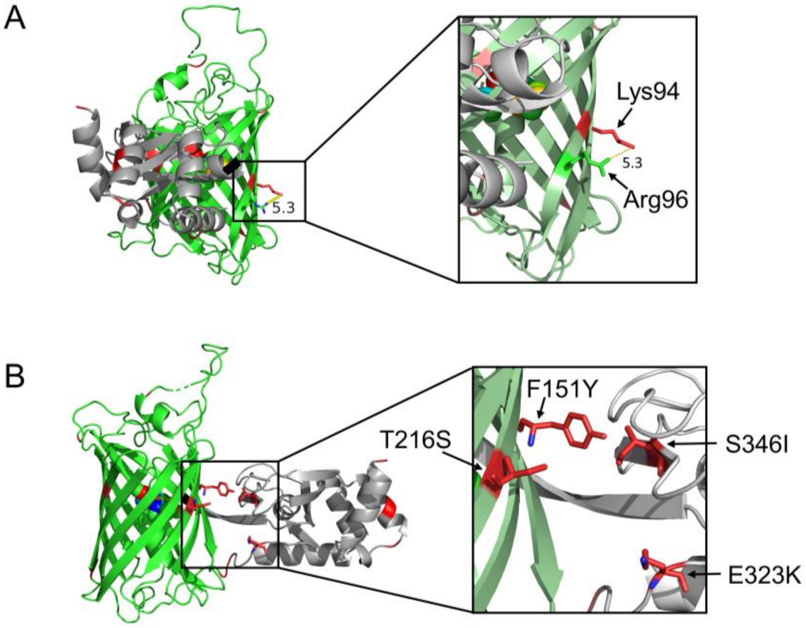

Supplementary Figure 4 Crucial mutations of G-Flamp2 and G-Flamp2b.

(A) Distance between the side chains of Arg96 and Lys94 in cpGFP.

(B) Mutations on interface, including F151Y in cpGFP, E323K and 346I in mlCNBD, and OPT mutation T216S.

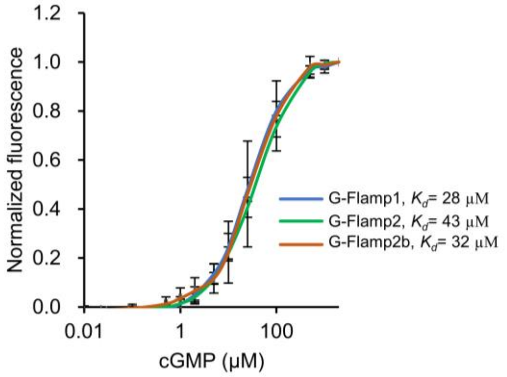

Supplementary Figure 5 Concentration-response curve of G-Flamp biosensors to cGMP.

Binding titration curves of G-Flamp biosensors to cGMP (500 μM) in HEPES buffer (pH 7.15). The data were fitted by a sigmoidal binding function to extract the dissociation constant K_d. Data are presented as mean ± standard deviation (SD) from three independent experiments.

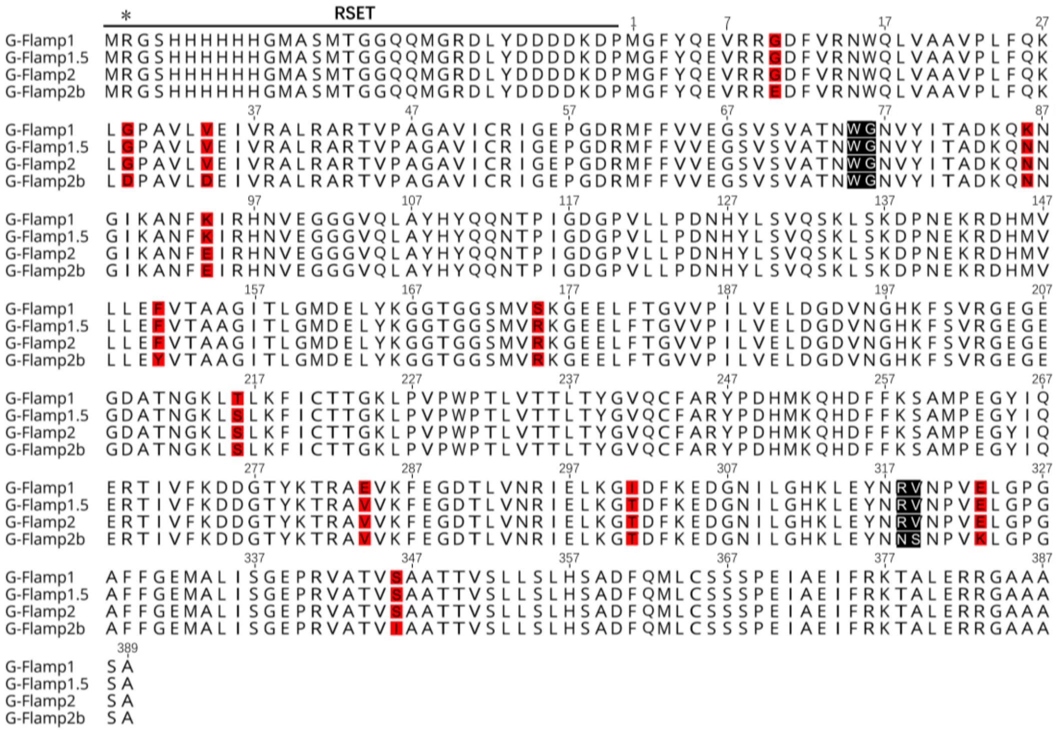

Supplementary Figure 6 Sequence alignment of G-Flamp2/2b to its parent G-Flamp1.

Sequence alignment of full-length G-Flamp2/2b to its parent G-Flamp1 and middle variant

G-Flamp1.5. Number 1 to 74 and 320 to 389 amino acids are sensing domain, number 77 to 317 amino acids is cpGFP. Linkers are highlighted in black. Mutations are highlighted in red. Note the second amino acid Arg in G-Flamp biosensors was deleted for mammalian expression.

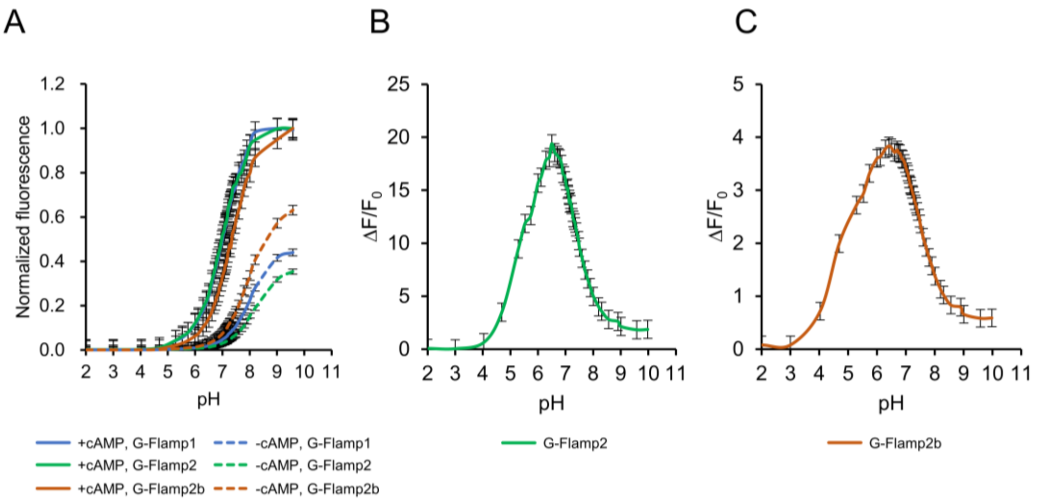

Supplementary Figure 7 pH-dependent properties of purified G-Flamp biosensors.

(A) pH-dependent fluorescence intensity changes of purified G-Flamp sensors with 500 µM cAMP (solid lines) and without 500 µM cAMP (dotted lines).

(B) (C) pH-dependent dynamic range changes of purified G-Flamp2 and G-Flamp2b.

The fluorescence intensities excited by 488 nm light were normalized to their maximum intensities, respectively. Data are presented as the mean ± standard deviation.

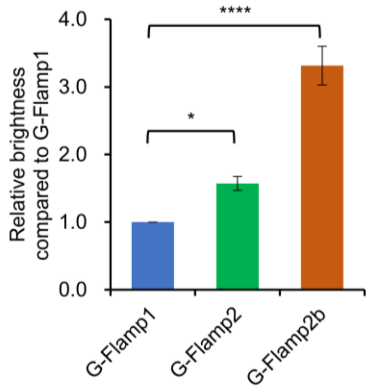

Supplementary Figure 8 Relative brightness of G-Flamp biosensors in HEK293T cells.

Relative brightness of G-Flamp1, G-Flamp2 and G-Flamp2b in resting HEK293T

cells measured using a plate reader (excited at 480 nm). The IRES-mCherry-CAAX gene was attached downstream of the green sensors and the red fluorescence intensity was used to calibrate the sensor's brightness. Data are presented as mean ± standard deviation (SD) from three independent experiments. *Significantly different from G-Flamp1, * $p < 0.05$, ****$p < 0.0001$ as measured by One-way analysis of variance (ANOVA) with post hoc Tukey's test.

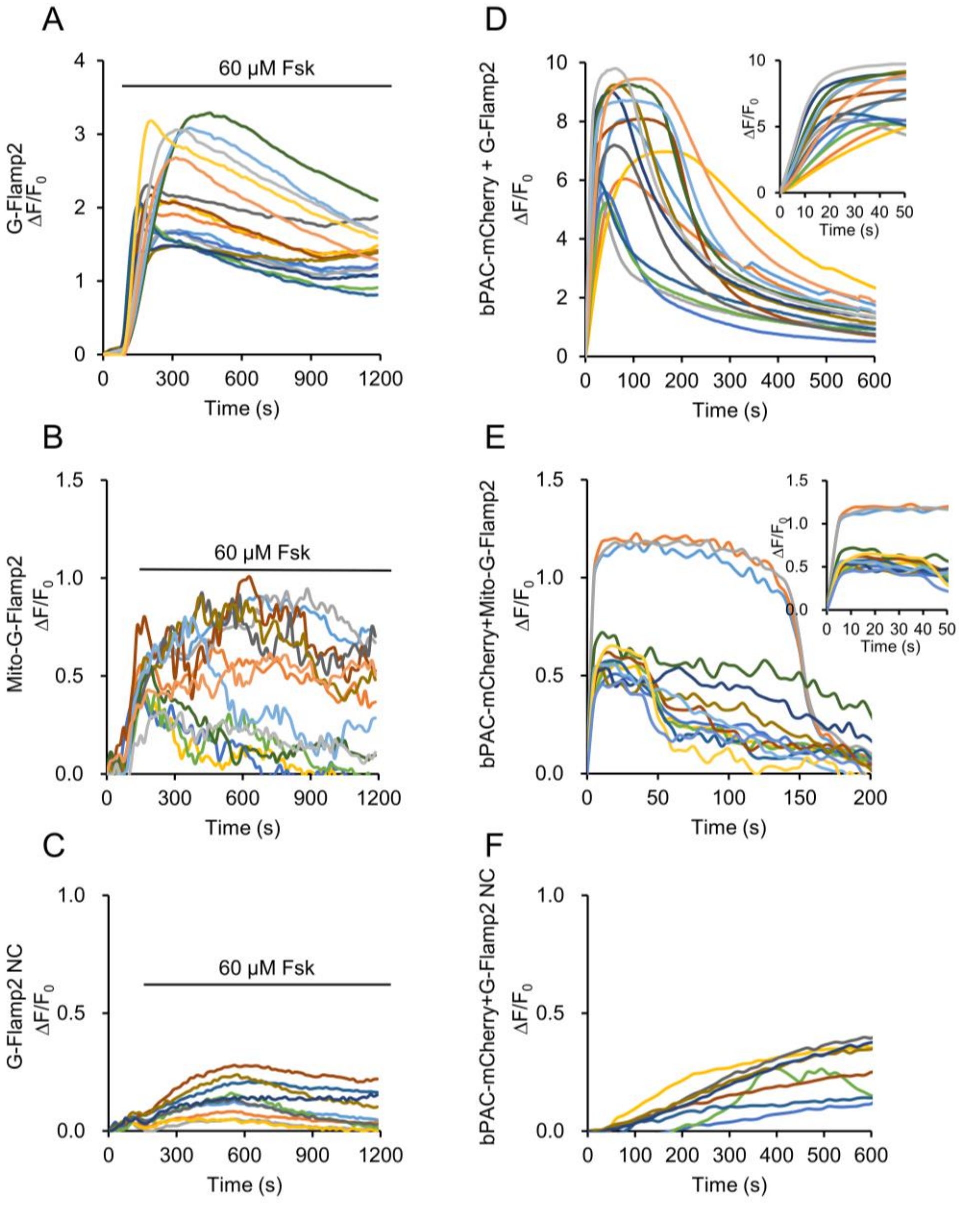

Supplementary Figure 9 cAMP produced in cytosol by Forskolin or bPACm reached the mitochondrial matrix.

(A) (B) and **(C)** Representative traces of $\Delta F/F_0$ in different cells were shown upon 60 μM Forskolin (added at 90 s). n = 16, 13 and 10 cells for GF2, Mito-GF2 and GF2 NC from 3 cultures for each sensor.

(D) (E) and **(F)** Representative traces of $\Delta F/F_0$ in different cells were shown with the

activation of bPACm. n = 15, 14 and 8 cells for GF2, Mito-GF2 and GF2 NC from 3 cultures for each sensor.

HeLa cells transiently transfected with GF2, Mito-GF2 and GF2 NC, images were taken after 48 hours transfection.

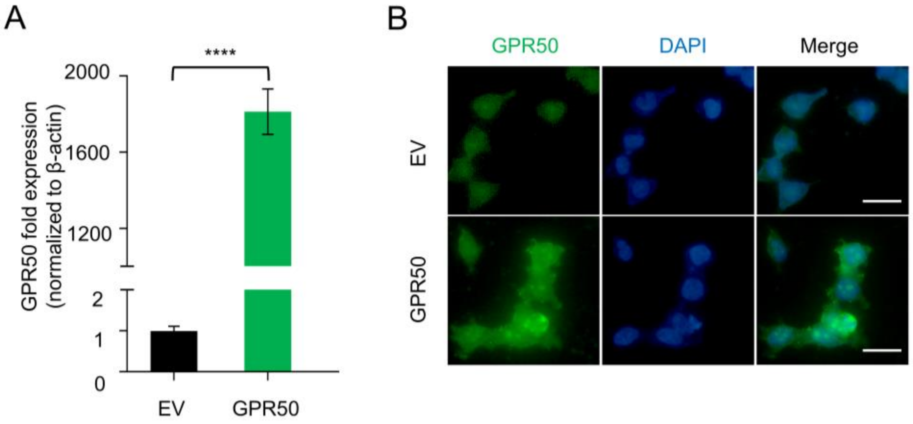

Supplementary Figure 10 GPR50 overexpressing cell line construction.

(A) RT-qPCR analysis of relative GPR50 gene expression in Empty Vector-293T (EV) or GPR50-293T (GPR50).

(B) GPR50 protein expression in Empty Vector-293T or GPR50-293T detected by immunofluorescence staining. An anti-GPR50 antibody recognizing the C-terminal of GPR50 was used as primary antibody. Values in bar graphs are means ± SEM. *Significantly different from EV, ****$p < 0.0001$ as measured Student *t*-test. (Scale bars, 25 μm)

Supplementary Table 1 Biophysical and biochemical properties of purified G-Flamp2.

Sensors	Ex/Em (nm), apo.	Ex/Em (nm), sat.	K_d (μM)	pka, apo.	pka, sat.	EC, apo. ($M^{-1}cm^{-1}$)	EC, sat.	φ, apo	φ, sat.
G-Flamp2	499/513	491/510	1.9±0.17	8.50	6.97	5189	45459	0.38	0.33
G-Flamp2b	499/513	491/510	3.2±0.42	8.56	7.35	12528	33760	0.39	0.37

Supplementary Table 2 Specificity of current cAMP indicators

Sensor	K_d[c] for cAMP (μM)	K_d for cGMP (μM)	specificity[d]	*Ref.*
mlCNBD-FRET[a]	0.07	0.5	7.1	[1]
cAMPr[b]	1	No response to 1 mM cGMP	n.d.	[2]
Flamindo2[b]	3.2	22	6.9	[3]
cADDis[b]	10-100	n.d.	n.d.	[4]
Pink Flamindo[b]	7.2	94	13.1	[5]
R-FlincA[b]	0.3	66	22	[6]
G-Flamp1[b]	2.2	30.1	13.7	[7]

[a]FRET-based cAMP indicator. [b]Single-FP cAMP indicators.

[c]Dissociation constant. [d]K_d ratio of cGMP/cAMP. n.d.: not determined

1. Mukherjee, S., et al., *A novel biosensor to study cAMP dynamics in cilia and flagella.* Elife, 2016. **5**.
2. Hackley, C.R., E.O. Mazzoni, and J. Blau, *cAMPr: A single-wavelength fluorescent sensor for cyclic AMP.* Sci Signal, 2018. **11**(520).
3. Odaka, H., et al., *Genetically-encoded yellow fluorescent cAMP indicator with an expanded dynamic range for dual-color imaging.* PLoS One, 2014. **9**(6): p. e100252.
4. *cADDis: Live Cell cAMP Assays*. Available from: https://montanamolecular.com/live-cell-camp-assay-caddis/.
5. Harada, K., et al., *Red fluorescent protein-based cAMP indicator applicable to optogenetics and in vivo imaging.* Sci Rep, 2017. **7**(1): p. 7351.
6. Ohta, Y., et al., *Red fluorescent cAMP indicator with increased affinity and expanded dynamic range.* Sci Rep, 2018. **8**(1): p. 1866.
7. Wang, L., et al., *A high-performance genetically encoded fluorescent indicator for in vivo cAMP imaging.* bioRxiv, 2022: p. 2022.02.27.482140.
